# A review on monitoring orthodontic treatment: from traditional approaches to innovative point-of-care tools

**DOI:** 10.3389/fdmed.2026.1928953

**Published:** 2026-09-24

**Authors:** Ananya Nair, Prajwal Shetty, M. S. Ravi

**Affiliations:** Department of Orthodontics and Dentofacial Orthopaedics, Nitte (Deemed to be University), AB Shetty Memorial Institute of Dental Sciences (ABSMIDS), Mangalore, Karnataka, India

**Keywords:** molecular diagnostics, orthodontic treatment monitoring, point-of-care (POC) diagnosis, point-of-care diagnostics, salivary biomarker

## Abstract

Orthodontic treatment is performed to treat malocclusions by improving oral functions, tooth alignment, and facial esthetics that impact the quality of life of patients significantly. Nevertheless, various factors like complexity of malocclusions, patient compliance, and dental hygiene can influence treatment outcomes. Traditional diagnostic methods such as clinical evaluation, radiography techniques, and dental casts have played a major role in orthodontics and have remained a standard diagnostic method in dentistry to date. On the other hand, these methods are associated with major disadvantages like radiation exposure and invasive procedures. To address these limitations, recent diagnostic methods have identified various biomarkers in gingival crevicular fluid and saliva as non-invasive diagnostic methods. These biomarkers can help clinicians in planning orthodontic treatment at the optimal time and determine the appropriate initiation of the treatment. Point-of-care diagnostic tools such as lateral flow assays, Enzyme-Linked Immunosorbent Assay, Clustered Regularly Interspaced Short Palindromic Repeats and microfluidic devices, and wearable technologies are emerging as promising alternatives for orthodontic treatment monitoring by offering rapid, cost-effective, and non-invasive alternatives to traditional methods. Recent developments such as incorporation of artificial intelligence and machine learning into orthodontic software attempt to improve the overall monitoring of orthodontic treatment. This review aims to present various traditional and innovative methods that have shown promising outcomes in research, along with insights into emerging areas that serve as potential game changers in orthodontic treatment monitoring.

## Highlights

- Traditional methods like radiography and clinical examination are slowly being replaced by advanced methods like biomarker-based and technology-driven diagnostic approaches in the field of orthodontic treatment monitoring.
- Translational research, where conventional methods are developed along with real-time monitoring techniques like wearable sensors and lab-on-a-chip technologies, will enable lab-to-bedside care.
- Integrating innovative diagnostics into orthodontic practice can reduce treatment time, improve patient compliance, and enhance clinical outcomes, marking a shift toward more efficient, tech-assisted oral healthcare.

## Introduction

Malocclusions are a most commonly occurring oral health concern that influences an individual's quality of life (1). To treat this, orthodontic procedures are performed that improve tooth alignment, oral function, and facial profile, contributing to the overall confidence of a person in a social and professional environment (2). However, for better outcomes, it is necessary to track the progress using various conventional and modern methods. Nevertheless, challenges such as patient compliance, oral hygiene, and the complexities in malocclusions remain challenging (3). Ideally, screening methods like clinical evaluation and radiographic techniques are widely used to monitor orthodontic treatment. But there is still room for discussion on the diagnostic approach of radiography and other techniques in orthodontic treatment (4).

Gingival crevicular fluid (GCF) and saliva are valuable indicators in orthodontic treatment monitoring, as they reflect changes in periodontal and bone health, thereby helping to monitor orthodontic treatments for better outcomes (5). Because of the increased levels of specific biomarkers, saliva is a preferred sample over GCF for assessing periodontal health. Studies by Toczewska et al (6) and Katsiki et al. (7) highlighted that saliva contains increased levels of matrix metalloproteinases (MMPs) and other proteolytic enzymes, which correlate with the severity of periodontal disease, while GCF shows lower concentrations of these biomarkers in inflamed tissues. The major advantage of saliva includes the non-invasive nature of sample collection, which helps in easier orthodontic treatment when compared with GCF, which is an invasive method of sample collection (8).

A biomarker is a substance that serves as an indicator of normal biological processes, diseases, and a response to treatment interventions. Many biomarkers have been discovered in GCF and saliva for the monitoring of orthodontic treatment, including Interleukin-1 beta (IL-1β), osteoprotegerin (OPG), and receptor activator of nuclear factor kappa-B ligand (RANKL), tumor necrosis factor-alpha, prostaglandin E2, osteocalcin, matrix metalloproteinases, acid and alkaline phosphatase (ALP), aspartate aminotransferase (AST), IL-1RA, interferon-gamma, and so on (9). A study by Abdul Wahab et al (10) showed that ALP, tartrate-resistant acid phosphatase (TRAP), and lactate dehydrogenase (LDH) were also present in saliva, which indicated saliva as a prospective source of biomarkers and therefore, a potential diagnostic tool during orthodontic tooth movement (11).

The standard method for monitoring orthodontic treatment is the use of radiography, which is widely practiced. However, this technique comes with disadvantages such as exposure to harmful ionizing radiation and can be expensive. However, to overcome these drawbacks, techniques that include three-dimensional (3D) images, such as a cone-beam computed tomography (CBCT) scan, were considered as an alternative method for treatment monitoring (12). Alternatively, techniques like Enzyme-Linked Immunosorbent Assay (ELISA), high-performance liquid chromatography (HPLC), Western blot, mass spectrometry (MS), spectrophotometry, polymerase chain reaction (PCR), and two-dimensional sodium dodecyl sulfate-polyacrylamide gel electrophoresis (2DE-SDS-PAGE) play a major role in the diagnosis of various biological markers (13). However, it becomes very challenging to implement these methods because of their cost, time consumption, and the need for advanced equipment. To overcome these drawbacks, a potential diagnostic method, called lateral flow assays (LFAs), can present as a promising alternative for monitoring orthodontically induced inflammatory root resorption by detecting specific biomarkers in oral fluids, providing a non-invasive and rapid diagnostic option that can enhance patient care compared with traditional methods. Overall, although LFAs improve accessibility and reduce the need for invasive procedures, their sensitivity and specificity must be further optimized for broader clinical application (14). Nevertheless, there are new techniques like the microfluidic and lab-on-a-chip method that include microfluid technology to analyze biomarkers with low sample requirements (15). In addition, wearable devices that help provide real-time feedback to evaluate changes in alignment in the jaw would enhance treatment compliance (16). Recent developments in the incorporation of artificial intelligence (AI) in oral healthcare have played a major role in orthodontics that could help identify treatment outcomes and patient non-compliance (17). Development of these technologies can help reduce frequent in-office visits, improving patient experience and enabling orthodontists to monitor treatment progress.

The aim of this review is to outline the different traditional diagnostic methods and the newly developed techniques by using specific biomarkers, the type of samples used for identification of biomarkers that would help monitor orthodontic treatment, and provide innovative point-of-care tools to improve the patient’s dental care and enhance the overall healthcare system.

## Conventional methods for orthodontic monitoring

Despite the emergence of advanced technologies, conventional methods are still practiced because of their reliability, accessibility, and affordability. However, conventional methods completely rely on in-office visits and physical examinations to evaluate orthodontic treatment monitoring (18). The various conventional methods are described in the following section. Figure 1 represents different conventional and advanced diagnostic methods for the monitoring of orthodontic treatment.

**Figure 1:**
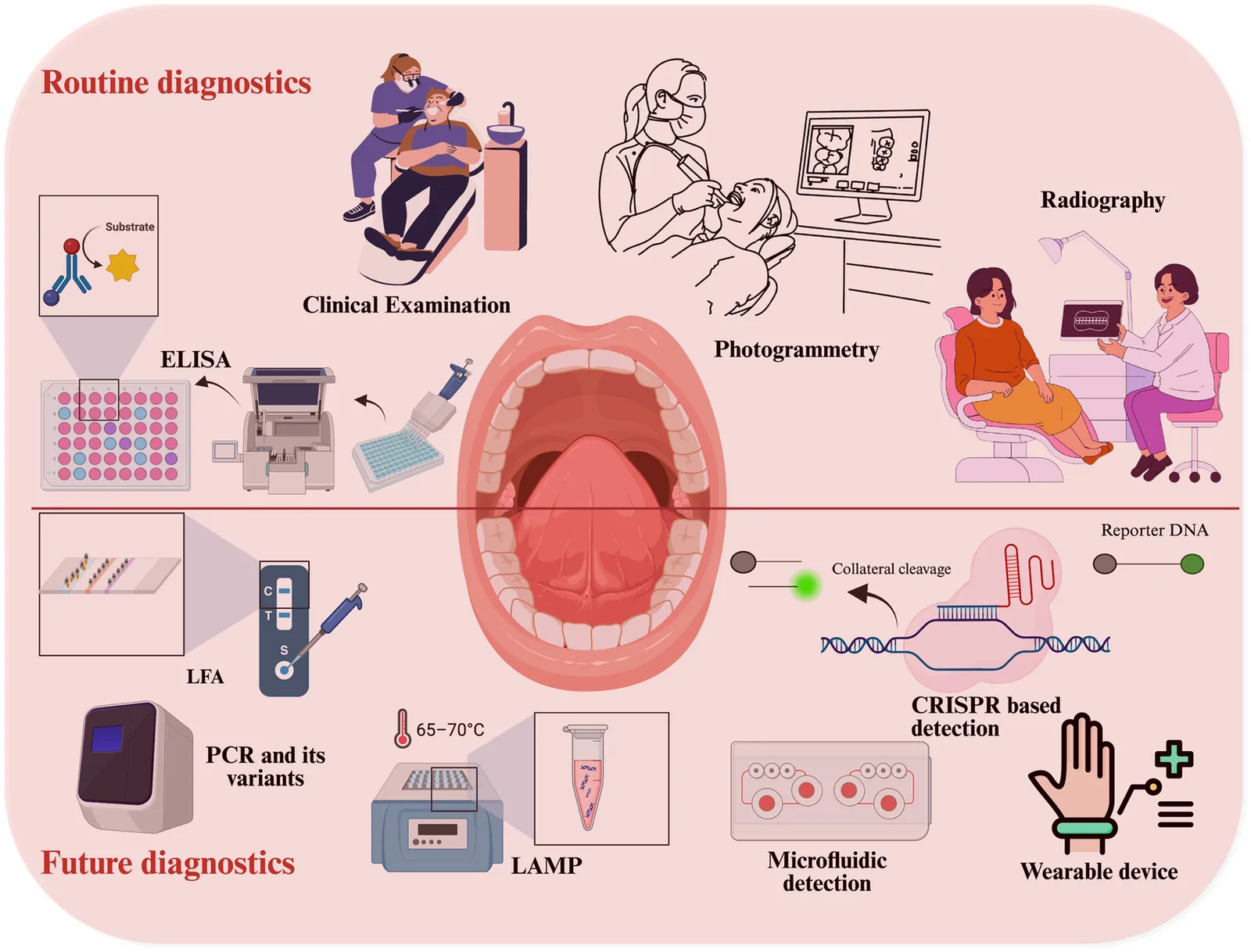
Diagnostic methods for orthodontic treatment monitoring.

## Clinical examination

Clinical examination is the most commonly performed orthodontic treatment monitoring method that includes both extraoral and intraoral examinations (19), providing a detailed image of the patient's dental alignment and occlusal relationships that are crucial for orthodontists to plan the treatment (20). A systematic review meta-analysis by Gimenez et al. (21) demonstrated that visual inspection indicated a higher rate of accuracy with a tendency toward a high value of specificity when compared with sensitivity, and hence, it is recommended in dental practice to follow this method and avoid any adjunctive techniques.

## Radiographic techniques

In the initial phase of diagnosis, radiographic techniques are considered a cornerstone because of their precise diagnosis. Several techniques that include cephalometric analysis, panoramic radiography, and periapical radiographs help evaluate the maxillofacial skeleton, dentition, soft tissues, orientation of the impacted teeth, growth and development, tooth movement, and so on. However, according to studies, the usage of these techniques is still debatable due to their high radiation exposure and the need for multiple X-rays (4, 22).

## Dental casts and study models

Dental casts are considered a gold standard for monitoring orthodontic treatment, along with imaging technologies, providing precision and reliable details about patients’ dental arches and teeth position (22). Alginate is used to create an impression of the patient's dentition, which is then replicated using plaster models. They are cost-effective and easily manufactured but are prone to breakage, tearing, and fracture over time and involve storage expenses. However, intraoral scanners used in digital models have replaced the conventional plaster model method by addressing these disadvantages. Digital models have improved orthodontic treatment planning, making the process more efficient and eliminating many of the limitations associated with traditional methods (23).

## Periodontal assessments

Throughout the orthodontic treatment phase, it is necessary to conduct a periodontal evaluation from diagnosis to post-treatment evaluation. Orthodontic appliances that create pressure and friction on the gums can lead to localized reaction in the gingiva, along with plaque buildup, which can create a challenging environment to maintain oral hygiene, resulting in the incidence of gingivitis and further complicating the orthodontic treatment process (24). Gingivitis and periodontitis are diagnosed with a periodontal probe that measures the depth of the pockets between the gums and the teeth and can act as an indicator of the severity of the infection (25).

A periodontist and an orthodontist are required to work hand in hand to evaluate periodontal concerns and decide on the most appropriate orthodontic treatment monitoring to improve the overall treatment outcomes.

## Photogrammetry

The American Society of Photogrammetry defines photogrammetry as the art, science, and technology of obtaining reliable information about physical objects and the environment, through the process of recording, measuring, and interpreting imagery and digital representations of energy patterns derived from non-contact sensor systems (26). This technology transforms small objects into 3D representations with similarity to the original object, preserving their size and shape. It is an important tool that helps develop digital dental models from the conventional plaster ones. In addition, due to its affordability, it is a better alternative to traditional scanning techniques (27).

## Advanced methods for orthodontic monitoring

The development of technologies in recent years has impacted the clinical workflow of dentists, from the process of diagnosis to treatment approaches, enhancing therapy, efficiency, and effectiveness (2). 3D imaging systems are the pillars of dental applications that have played a key role in enhancing accuracy in diagnosis and treatment planning. In this regard, computed tomography (CT) is commonly performed when detailed three-dimensional imaging is required, despite it being expensive and its high exposure to radiation. Compared with traditional CT applications, the advancement of CBCT provides significant benefits compared with the conventional method (28). CBCT is a radiographic technique that uses a cone-shaped source beam of ionizing radiation along with a two-dimensional detector. It provides a multidimensional image of the impacted teeth, along with the assessment of the roots of the impacted and adjacent teeth (29). Portelli et al. stated that CBCT plays a key role in diagnosis and treatment planning but requires more evidence to prove greater reliability (30). However, CBCT has limitations, including exposure to radiation, high cost, and limited ability to represent the internal structure of soft tissues or detect soft tissue lesions (29).

## AI and machine learning

Over the years, there has been a significant increase of artificial intelligence technology in the clinical use of orthodontic workflow and is considered the future of dental applications. Artificial intelligence–based software systems have a range of applications in diagnosis and treatment that involve patient communication and diagnosis to enhance treatment outcomes. The software systems employed in orthodontics use AI technologies based on machine learning, which implies that machines utilize the collection of raw data from a dataset in machine learning technology. Dental photographs and radiographs are analyzed by these software programs, providing guidance to orthodontists during 3D intraoral scanning. AI can make a great contribution to the dental industry by being cost-effective, requiring limited manpower and improving the diagnosis and treatment process (31–34).

## Point-of-care diagnostic tools

### Lateral flow assays

LFAs are widely used diagnostic devices noted for their quick results, ease of use, and cost-effectiveness. They are operated according to the principle of capillary action, where a liquid sample migrates along with a test strip and interacting with specific reagents to create a detectable signal in the form of color lines (35). In orthodontics, monitoring biomarkers like can provide information about tissue responses to orthodontic therapy during treatment. LFAs are investigated to assess the amount of inflammation by detecting IL-1beta in gingival crevicular fluid (36). The generation of LFAs that target particular biomarkers offers a quick, non-invasive method that can rapidly track patient responses, along with guiding personalized orthodontic treatments (37). In their studies, He et al. (38) developed a sensitive and specific disk-like lateral flow immunoassay strip by using green core–shell upconversion nanoparticles as a luminescence probe to identify biomarkers like MMP-8, 1L-1β, and TNF-α in GCF that showed high linear correlations for accurate clinical diagnostic methods. LFAs offer several distinct advantages that have made them indispensable in point-of-care diagnostics. They are inexpensive, easy to use, and provide rapid results without the need for trained personnel or sophisticated instrumentation. Despite these strengths, sensitivity remains a major challenge, particularly when detecting ultralow concentrations of analytes in complex biological samples. Issues such as variability in sample volume, pore blockage, and antibody inactivation compromise reproducibility and accuracy. Most LFAs provide qualitative or semiquantitative results, limiting their precision in clinical decision-making (39).

### Enzyme-linked immunosorbent assay

ELISA is a high-sensitivity immunoassay that is used to analyze saliva and gingival crevicular fluid samples to identify biomarkers of orthodontic tooth movement. However, when measured in lesser quantities of samples, it might fail to provide a greater standard curve because of the background activity and may provide similar values as for the blank (11).

Studies by Abdul Wahab et al. (10) evaluated total levels of proteins such as ALP, LDH, TRAP, and AST present in the saliva in GCF during orthodontic treatment monitoring. These studies showed no evident correlation between these proteins and the activity of enzymes, denoting the presence of a combination of active and inactive enzymes in GCF and saliva. Therefore, concluding an enzyme assay approach is advisable over ELISA.

Baeshen (40) evaluated defensin protein levels in saliva, emphasizing lower levels of defensins HBD-1, HBD-2, HNP-1, and HNP-2 in the saliva of patients wearing conventional lingual appliances compared with labial fixed appliances.

Inchingolo et al. (41) measured IL-17A, RANKL, and TNF-α levels in the treatment and control groups. The study showed increased levels of IL-17A and RANKL and decreased levels of TNF-α in the treatment group compared with controls after orthodontic force application. Direct ELISA is quick and simple but limited by low sensitivity, dependence on high-quality antibodies, restriction to single analyte detection, risk of background noise and cross-reactivity, a narrow dynamic range, and being time-consuming for large sample sets. These drawbacks must be weighed against its ease of use when selecting the appropriate assay format (42).

### LAMP

Loop-mediated isothermal amplification (LAMP), first developed in the year 2000 by a Japanese company, is a nucleic acid amplification technique that enables rapid and specific DNA amplification under constant temperature conditions (43).

This method utilizes four to six primers, which include two inner and two outer primers, that target a total of six distinct sequences on the gene of interest, hence enhancing specificity. The amplification runs at a constant temperature, during which the process is monitored in real time using turbidity measurement, fluorescence, or colorimetric indicators (43, 44). Unlike a PCR that requires advanced technical skills, the limitations of standardized protocols create a need for quicker, more sensitive, and efficient techniques like LAMP, which provides high sensitivity when compared with other molecular methods. This method is considered more efficient than PCR because of its capacity to produce billions of copies of fragments within an hour. LAMP has generated interest among the dental community, where it is used to determine its applications in dental practice over the frequently used molecular methods. Studies by Sangolli et al. (45) compared qPCR (real-time PCR) and conventional PCR with LAMP to detect *Porphyromonas ginigvalis* in 50 subgingival plaque samples, where LAMP was able to detect *P. ginigvalis* in 40 out of 50 samples, whereas qPCR and conventional PCR were able to detect *P. ginigvalis* in 38 and 33 samples. However, LAMP presents limitations: the primer design is complex and highly constrained, fluorophore–primer interactions can reduce reproducibility, and multiplexing for several biomarkers remains technically challenging with no standardized methodology. Moreover, many methods require careful optimization and have yet to be validated extensively in field settings, restricting their immediate translation into routine diagnostics (46).

### CRISPR

Clustered Regularly Interspaced Short Palindromic Repeats (CRISPR) technology has emerged as a revolutionary milestone in medical and biological science, specifically contributing to its applications in genetic and molecular biology. A CRISPR locus consists of a CRISPR array, having short and repetitive DNA sequences, enclosed with spacers. A leader sequence helps in adaptation and transcription, and cas genes that encode nucleases, called “cas proteins,” which utilize CRISPR-derived RNAs to identify and cleave foreign nucleic acid. To date, 93 different cas genes have been discovered, classified into 35 families (47, 48). Recent advancements in CRISPR have attracted significant interest in the field of dentistry, offering a promising paradigm in oral health through various applications. Malocclusions and dentofacial deformities caused by genetic mutations can represent a potential challenge for clinicians, as these genetic mutations cannot be altered. However, CRISPR technology can enable genome modifications in the future and detect and correct genetic mutations that are responsible for craniofacial malformations (49). In addition, this technique can play a key role in oral cancer by targeting oncogenes, providing a more precise approach compared with traditional methods. CRISPR can offer its applications in salivary diagnostics by detecting specific biomarkers in saliva, facilitating early diagnosis of dental conditions (50). However, this technique can be expensive and might require expert technical skills (51). Despite its transformative potential, the most pressing issue is the high frequency of off-target effects, which can introduce unintended mutations and compromise genomic stability (52).

### Microfluidic and lab-on-a-chip method

Microfluidic technology, also known as lab-on-a chip systems, manipulates tiny fluid volumes in carefully crafted microchannels to enable high throughput and real-time biomarker analysis (53). On a single chip, these devices combine laboratory processes like sample preparation, separation, and detection. Rapid and precise biomarker identification is essential for a variety of applications, including infectious diseases detection and cancer diagnostics (54). Advanced detection methods such as electrochemical and fluorescence-based systems have been incorporated into recent innovations to further improve sensitivity and accuracy (55).

One of the main benefits of microfluidic devices is their portability. They are ideal for point-of-care diagnostics and application in environments with limited resources because of their small size and low sample requirements. In addition, lab-on-chip solutions increase operating efficiency by reducing the need for substantial laboratory infrastructure (56). Also, these methods shorten the time between sample collection and outcomes, which is very advantageous in emergency or field settings (57). The key limitation of microfluidics in orthodontic monitoring lies in its insufficient resolution at the subcellular level. This restricts the ability to analyze fine cellular details such as morphology, movement, and cytokine secretion, which are essential for understanding junctional protein roles in mechanotransductive signaling. As a result, current insights into cellular interactions within microfluidic systems remain incomplete, highlighting the need for more advanced approaches to capture these intricate processes (58).

### Wearable devices

Orthodontic care has been transformed by the combination of wearable technology and orthodontic appliances, which enables ongoing tracking of patient compliance and treatment progress. To monitor patient adherence, early innovations focused on wearable sensors integrated into headgear or detachable appliances (59). Parafunctional habits like bruxism, jaw movement, and appliance use can all be tracked by wearable technology, including microelectronic sensors. For instance, these sensors provide real-time feedback to patients and orthodontists by monitoring wear time and identifying changes in jaw alignment (16). In addition, clinicians can improve treatment strategies and identify problems like inflammation or inappropriate force distribution by using wearable technology to gather data on gingival health and force application. The non-invasive characteristics of these devices have made them a desirable option for enhancing patient engagement and tracking compliance (36).

### Biomarker-based monitoring

Orthodontic tooth movement can cause an inflammatory reaction on both biochemical and structural fronts, which secretes certain biological biomarkers and inflammatory mediators, which play a key role in monitoring the treatment process. The various biomarkers that contribute to orthodontic treatment monitoring are listed in the following sections. Table 1 provides a list of biomarkers used in orthodontic treatment monitoring.

**Table 1:** Biomarkers in orthodontic treatment monitoring.

Biomarker	Type	Source	Role in orthodontics	Clinical relevance	Reference
IL-1β	Cytokine	GCF and saliva	Indicates bone remodeling and inflammatory response	Elevated during active tooth movement	(75)
TNF-α	Cytokine	GCF	Inflammatory mediator involved in osteoclastic activity	High levels suggest active resorption	(63)
MMP-8	Enzyme	GCF and saliva	Collagen degradation and extracellular matrix remodeling	Increased levels indicate periodontal breakdown	(6, 7)
ALP	Enzyme	GCF	Bone formation marker	Higher levels indicate active bone deposition	(10)
RANKL	Protein	GCF	Regulates osteoclast activity	High levels linked to bone resorption	(62)
OPG	Protein	GCF	Inhibits RANKL, balancing bone resorption	Low levels may indicate excessive resorption	(9)
ALP		GCF and saliva	Bone formation–related markers	Upregulation corresponds to heightened osteoblastic activity and extracellular matrix deposition during alveolar bone remodeling on the tension side	(76)
TGFβ		GCF and saliva	Bone formation–related markers	Upregulation corresponds to heightened osteoblastic activity and extracellular matrix deposition during alveolar bone remodeling on the tension side	(76)
IL-6		Saliva	Inflammatory and resorptive	Bone resorption	(76)

### Salivary biomarkers

Saliva presents as a non-invasive alternative approach that can help evaluate the effectiveness or potential side effects of the treatment. Saliva is a complex fluid that has contributions from GCF, salivary glands, blood, the oral microbiome, and the respiratory system. Saliva is considered to be a more reliable sample than GCF for assessing periodontal health, as it contains higher concentrations of distinct biological markers, while GCF shows lower concentrations of biomarkers (5). Saliva not only has a non-invasive nature of sample collection but also helps indicate the individual's overall physiological condition, which makes it easier to monitor the oral health of the patient over time. Enzymes like proteases, amylase, carbonic anhydrase, and catalase are also identified in saliva. Because of the forces applied on the periodontal ligament (PDL) that stimulate the release of cytokines, enzymes and inflammatory mediators can act as biochemical indicators. Bone resorption may lead to the secretion of inflammatory mediators, such as prostaglandin E (PGE) and interleukin (IL)-1, which interact with bone cells and serve as important biomarkers for inflammation during the treatment phase (60, 61).

Studies by Flórez-Moreno et al. (62), examined RANKL and OPG levels of 20 orthodontic patients in unstimulated saliva samples. The results demonstrated that RANKL levels had significantly increased, while they observed a significant decrease in OPG levels, suggesting that variations in RANKL and OPG concentration and their ratio might be correlated to the various phases of orthodontic tooth movement. Abdul Wahab et al. (10) analyzed the enzyme activity in saliva during orthodontic tooth movement with self-ligating brackets. They indicated that ALP, AST, and tartrate-resistant acid phosphatase are prospective biomarkers for orthodontic treatment monitoring. Salivary IL-1β and TNF-α values were calculated by Saadi and Ghaib, and they highlighted that highest values were found at 1 h after the placement of a round nitinol wire during the treatment, which declined after 2 weeks (63). A systematic review by Rodrigues et al. (64) analyzed 7,038 records through electronic search, out of which only 12 met the eligibility criteria and were included in a qualitative analysis. According to this review, increased levels of enzymes like ALP, LDH, MMP8, and MMP9 were noticed. Mediators like sRANKL, OPG, and IL-1*β* showed fluctuations at the various treatment phases (64).

### GCF analysis

The inflammatory changes caused by the orthodontic forces can result in bone remodeling that can lead to the secretion of GCF as an inflammatory exudate in addition to various cytokines, electrolytes, antigens, and proteins. GCF is derived from serum, host inflammatory cells, periodontal structural cells, and oral bacteria. The orthodontic force can cause an inflammation of periodontal tissues that could lead to the activation of cytokines, which include matrix metalloprotein, IL-1β, and tumor necrosis factor-alpha (TNF-α). OPG and osteopontin (OPN) serve as biochemical markers of bone metabolism. In addition, there are increased levels of RANKLP and decreased levels of OPG that are likely to originate from GCF. Although GCF is a very useful diagnostic biomarker in orthodontics, GCF collection can be limited in routine clinical practice due to its cumbersome nature, reflective of the scarcity of data available to reach full diagnostic utility (8, 9, 65–67).

A study by Rao et al. (68) compared the levels of interleukin-6 in GCF and periodontal ligament during and before orthodontic treatment. The study observed increased levels of IL-6, and these levels were found to be statistically significant (69). A study by Chelărescu et al. (70) observed increased levels of interleukin (IL)-1β in an ELISA test in a group of 20 subjects aged between 11 and 28 receiving orthodontic treatment; the study highlighted that the periodontal tissues of adolescent patients were more responsive to the treatment than those of young adults. A study by Castroflorio et al. (71) observed that there was a rise in the concentration of bone modeling and remodeling mediators like IL-1β, RANKL at the pressure points, transforming growth factor (TGF)-β1, and OPG at the tension sites in patients undergoing orthodontic treatment in which aligners were used, thus highlighting that these biomarkers play a key role in orthodontic treatment monitoring. Tian-yi et al. (72) observed reduced concentration of IFN-*γ*+ Th1 cells in patients with grade C periodontitis, thereby controlling the local and systemic levels of inflammation.

### Serum biomarkers

The general dental growth status of a person undergoing orthodontic treatment describes the skeletal maturity level by the presence of biomarkers in serum. Studies evaluated the serum levels for osteocalcin, bone ALP, and serum carboxy-terminal telopeptide, and it was observed that elevated levels of markers were present in the pubertal age group. However, there was a decrease in the markers with sexual maturation and advancing bone age, highlighting the correlation to bone mineral density (73). However, due to the invasive nature of the sample collection, a line is drawn when compared with the other invasive techniques.

## Future perspective and challenges

### Advances in real-time monitoring technologies

Significant advancements in orthodontics have been brought about by developments in real-time monitoring technologies. For instance, precise treatment adjustments are made possible by force-sensing orthodontic applications, which are able to measure stress distribution and detect occlusal forces (53). These devices continuously measure bite force using cutting-edge materials like piezoelectric sensors, which are essential for malocclusion correction and tooth alignment.

Wearable biosensors with cloud and Bluetooth integration have also improved real-time data analysis. Patients and clinicians can view treatment progress and make prompt decisions, thanks to the direct data transmission from these devices to orthodontic applications. The chance of treatment delays or complications is greatly decreased by this real-time monitoring capability (37).

### Integration of AI and wearable devices in routine practice

Artificial intelligence is playing a transformative role in orthodontics by integrating wearable devices into routine practice. Large datasets produced by wearable technology can be analyzed by AI-driven algorithms, which can also spot treatment patterns and anticipate possible problems like patient non-compliance.

AI-powered applications, for example, can improve remote consultations in adherence facilities and send out customized treatment reminders. Frequent in-office visits are being replaced by virtual orthodontic monitoring platforms that evaluate patients’ submitted images using artificial intelligence. In addition to increasing convenience, this wearable technology and AI combination guarantees ongoing patient–clinician interaction (57). However, the clinical implementation of AI faces challenges, including the need for robust external validation and assessment of model generalizability across diverse patient populations. Ethical concerns related to patient privacy, informed consent, data security, algorithmic bias, and transparency must also be addressed before widespread clinical adoption.

Table 2 provides insights on different diagnostic methods for monitoring dental treatment.

**Table 2:** Diagnostic technologies and methods for monitoring dental treatment.

Method	Limit of detection/sensitivity and specificity	Time taken	Advantages	Disadvantages	References
LAMP	Sensitivity −94.87% Specificity −90.90%	4–6 h	Quick, accurate, and reliable, suitable for point-of-care testing	Lower sensitivity and delayed amplification	(43)
Wearable devices	Sensitivity depends on sensor type; moderate specificity	Continuous monitoring	Real-time tracking of jaw movements and compliance assessment	May be uncomfortable, requires patient compliance	(59)
Microfluidics	High sensitivity and specificity for biochemical markers	1–3 h	Minimal sample required and rapid biochemical analysis	High cost, requires specialized equipment	(57, 77)
Lab-on-a-chip	Sensitivity >90%, Specificity −95%	2–4 h	Portable, efficient, multiplexing capability	Requires trained personnel, potential cross-contamination	(78)
Radiographic techniques (CBCT and panoramic X-ray)	High specificity and moderate sensitivity	Minutes to hours (depending on analysis)	High-resolution imaging, useful for structural assessment	Radiation exposure, costly	(79, 80)
Clinical examination	Varies depending on examiner experience	Instant	Non-invasive and cost-effective	Subjective, requires expertise	(20)
LFA	Low sensitivity, moderate specificity	2–4 h	Simple, rapid, and low cost	Sensitivity and specificity	(81)
ELISA	Sensitivity varies; specificity can be influenced by cross-reactivity	2–4 h	Can be semiquantitative; useful for biomarker detection	Potential cross-reactivity; lower sensitivity than molecular methods	(77)
CRISPR	Sensitivity −80%, Specificity −100%	2–4 h	High specificity and rapid detection of genetic markers	Lower sensitivity than molecular assays	(82, 83)

### Barriers to widespread adoption

Wearable technology integration in orthodontics requires a large outlay of funds. These sophisticated devices are costly to develop, produce, and maintain, which frequently raises the cost of patient care. The cost of wearable technology remains one important obstacle, particularly in environments with limited resources (35). Wearable device applications function inconsistently because of a lack of standardization in data management and device design. Uniform treatment outcomes are hampered by variations in device calibration, data collection techniques, and analysis protocol. For broad adoption, industry-wide standards and guidelines must be established (74). Comprehensive training for orthodontists and their staff is necessary for successful integration of wearable technologies. A lot of resources may be needed for practices to adopt the required new skill sets to comprehend device functionality and interpret data that have been collected. For this wearable technology to be used effectively, these training gaps must be filled (37).

## Conclusion

Despite growing research on orthodontic treatment monitoring through various traditional and innovative approaches, which helps in the early identification and subsequent management of treatment, there remains a large scope to evaluate novel biomarkers and innovative methods that are specific to orthodontic treatment. Our review concludes that simultaneous utilization of innovative and traditional methods with AI may aid in better treatment diagnosis and management. Table 3 explains the advantages and disadvantages of different diagnostic methods.

**Table 3:** Advantages and limitations of different diagnostic methods.

Method	Clinical applicability	Advantages	Limitations	Invasiveness	Cost	Evidence
Clinical examination	Routine orthodontic assessment	Simple, accessible, reliable, and high specificity	Subjective, limited sensitivity, and clinician-dependent	Non-invasive	Low	High
Radiography (Ceph, CBCT)	Diagnosis and treatment planning	Precise imaging and 3D visualization	Radiation exposure, expensive, and limited soft tissue detail	Minimally invasive (radiation)	Moderate–high	Moderate–high
Dental casts/study models	Arch and tooth position evaluation	Gold standard, precise, and cost-effective	Fragile, storage issues; digital models costly	Non-invasive	Low–moderate	High
Periodontal assessment	Gingival health monitoring	Direct measure of periodontal status	Requires specialist input and invasive probing	Minimally invasive	Low	Moderate
Photogrammetry/digital models	Digital replication of dentition	Affordable, accurate 3D models	Limited resolution and requires software	Non-invasive	Moderate	Emerging
LFAs	Point-of-care biomarker detection	Rapid, inexpensive, and non-invasive	Lower sensitivity and qualitative results	Non-invasive	Low	Emerging
ELISA	Biomarker quantification in saliva/GCF	High sensitivity and an established lab method	Time-consuming, single analyte detection, and background noise	Minimally invasive (GCF)	Moderate–high	High
LAMP	DNA amplification for biomarker detection	Rapid, highly sensitive, and efficient	Complex primer design and limited multiplexing	Non-invasive (saliva)	Moderate	Emerging
CRISPR-based assays	Genetic/molecular biomarker detection	High precision and mutation identification	Off-target effects, expensive, and technical expertise required	Non-invasive	High	Early stage
Microfluidics/lab-on-a-chip	Real-time biomarker analysis	Low sample requirement, portable, and efficient	Limited subcellular resolution	Non-invasive	Moderate–high	Emerging
Wearable devices	Patient compliance and treatment monitoring	Real-time feedback and enhances engagement	Limited biomarker detection and integration challenges	Non-invasive	Moderate–high	Emerging
AI/machine learning	Diagnostic support and treatment prediction	Cost-effective and improves accuracy	Dependent on dataset quality and requires validation	Non-invasive	Moderate–high	Emerging
